# Developing and validating anti-ADA2 single-chain antibodies coupled to alkaline phosphatase for diagnosing pleural tuberculosis

**DOI:** 10.3389/fimmu.2025.1646134

**Published:** 2025-08-14

**Authors:** Maksym Skaldin, José M. Porcel, Urpo Lamminmäki, Silvia Bielsa, Andrey V. Zavialov

**Affiliations:** ^1^ International Center for Aging and Cancer (ICAC), Hainan Medical University, Haikou, China; ^2^ Turku Center for Biotechnology, University of Turku, Turku, Finland; ^3^ Joint Biotechnology Laboratory, University of Turku, Turku, Finland; ^4^ Pleural Medicine Unit, Department of Internal Medicine, Biomedical Research Institute of Lleida (IRBLleida), Arnau de Vilanova University Hospital, Lleida, Spain; ^5^ Department of Life Technologies, University of Turku, Turku, Finland; ^6^ Institute of Pediatrics, Guangzhou Women and Children's Medical Center, Guangzhou, Guangdong, China

**Keywords:** ADA2 (Adenosin deaminase 2), pleural ADA, pleural tuberculosis, pleural tuberculosis diagnosis, single chain (scFv), ADA2 ELISA

## Abstract

**Introduction:**

Adenosine deaminases ADA1 and ADA2 reduce adenosine concentrations, which regulate cellular immune responses to activation signals. It has been shown that ADA2 activity increases in the pleural fluid of patients with tuberculosis (TB).

**Methods:**

We engineered recombinant scFv-AP antibodies using phage display technology to select high-affinity binders against ADA2. These were incorporated into a sandwich ELISA, allowing for the precise measurement of ADA2 levels in pleural fluid.

**Results:**

The assay was tested on pleural samples from 41 patients with TB and 47 with non-TB effusions, including those with malignancies and parapneumonic effusions. Results showed that ADA2 concentrations were significantly higher in patients with TB than in other groups, and the ADA2-based assay exhibited improved diagnostic specificity (91%) compared with total ADA testing (76%). A cutoff of 300 ng/mL for ADA2 yielded a sensitivity of 98% and a negative likelihood ratio of 0.03, effectively ruling out TB when the result was negative.

**Discussion:**

The new ADA2 assay offers a simple, reliable, and more specific alternative for diagnosing pleural TB, with potential applications in other ADA2-related disorders.

## Introduction

Adenosine deaminases (ADAs) reduce adenosine concentrations by hydrolyzing adenosine to inosine. Large fluctuations in adenosine concentration occur during inflammatory conditions, stress, and tumor growth. Adenosine binds to adenosine receptors (A1 and A2 subtypes) expressed in nearly all cells and plays a crucial role in regulating a wide range of physiological processes in the body. Therefore, genetic defects leading to the inactivation of ADA1 and ADA2 can result in immune dysfunction. Recently, it was discovered that ADA2 is a lysosomal protein that may regulate the activation of plasmacytoid dendritic cells and macrophages (1, 2). Multiple studies have shown that estimating ADA activity in pleural fluid is a reliable biomarker for tuberculosis (TB) (3, 4). TB remains a global threat to public health, with more than ten million new cases estimated annually (5). Early diagnosis and effective treatment are essential for decreasing the substantial morbidity and mortality associated with TB. Pleural TB, which accounts for 35-50% of all extrapulmonary cases, is difficult to diagnose using conventional methods (6–8). ADA activity is high in TB pleural exudates (9, 10), and is mainly due to ADA2 secreted by TB-infected macrophages. Thus, ADA2 has been suggested for the diagnosis of TB pleural effusion (11). Clinical studies have also shown that the levels of ADA2 in biological fluids are increased in other pathophysiological conditions, such as HIV infection and breast cancer (12–14) and dramatically decreased in patients with ADA2 deficiency (DADA2) (15). This suggests that ADA2 activity could serve as an additional marker for the diagnosis and monitoring of these diseases. The assays currently used to measure total ADA activity are either based on the detection of ammonia, which is produced as a result of adenosine deamination (16, 17), or the detection of a peroxide ion, which is formed during the degradation of inosine to uric acid in the presence of purine nucleosidase and xanthine oxidase (10). The activity of ADA2 can be determined by adding erythro-9-(2-hydroxy-3-nonyl)adenine (EHNA), a potent inhibitor of ADA1 (11). Therefore, the difference between total ADA activity and the remaining ADA2 activity in the presence of EHNA is thought to be ADA1 activity. However, it is difficult to compare data on total ADA activity obtained from different clinical laboratories. This is because ADA1 and ADA2 have an approximately 100-fold difference in Km for adenosine and a distinct maximum pH for their respective ADA activities. Therefore, the measured ADA1 and ADA2 activities, as well as the total ADA activity, significantly depend on the pH of the buffer and adenosine concentration used in the assay. Indeed, the activity of ADAs in the plasma and pleural fluid of healthy controls varies significantly (12, 13, 18). Additionally, each available assay has its limitations, including low sensitivity, limited sample storage times, absence of internal standards, or high cost and complexity of commercial kits. Sample quality is also critical for the accurate determination of ADA activity using colorimetric assays. In addition, contamination of biological samples with lysed red blood cells or bacteria, a source of ADA1, may lead to a false increase in total ADA and ADA1 activities. Therefore, this study aimed to develop a simple assay for the quantitative determination of ADA2 in biological samples using ADA2 standards with known concentrations. We generated anti-ADA2 single-chain antibodies fused with alkaline phosphatase to determine the concentration of ADA2 in clinical samples. The assay was used to differentiate between tuberculous and non-tuberculous exudative pleural effusions, and the results of the analysis were compared with those of the total ADA activity assay.

## Materials and methods

### Patients

After obtaining approval from the local ethics board (CEIC 1868), 41 patients with pleural TB and 48 with non-TB effusions were recruited from a prospectively maintained database and biobank (IRBLleida Biobank B.0000682) at the University Hospital Arnau de Vilanova (Lleida, Spain). An elevation of pleural fluid ADA levels >35 U/L (the diagnostic cutoff for TB in our center) in at least 20% of the non-TB samples was established as a prerequisite. This criterion ensured an adequate number of participants with non-tuberculous effusions and elevated total ADA levels in pleural fluid, thereby accurately reflecting clinical reality.

### Diagnostic criteria

A definitive diagnosis of TB effusion was made if auramin staining or cultures (either in solid or liquid media) of pleural fluid, sputum, or pleural biopsy specimens were positive or if the latter showed granulomas in the parietal pleura. TB was considered probable in patients with lymphocytic exudates from the first or subsequent thoracentesis, high pleural fluid ADA levels (>35 U/L), negative cytological examinations, and clearance of effusion with anti-TB therapy. If malignant cells were detected on cytological examination of the pleural fluid or biopsy samples, the effusion was categorized as malignant. Parapneumonic effusion was defined as any effusion associated with bacterial pneumonia that was either cured with antibiotics (uncomplicated) or required chest tube drainage (complicated). Other causes of pleural effusion were determined using well-established clinical criteria.

### ADA measurement

Pleural fluid samples obtained during thoracentesis were collected in 5 mL sterile heparinized tubes for immediate routine biochemical analysis, including ADA. Total ADA activity was determined using an automated spectrophotometric method (Roche Diagnostics, Barcelona, Spain).

### Phage display selection of scFv antibodies

A. Coating beads with ADA2 protein

Tosyl-activated M-280 Dynabeads (Invitrogen Life Technologies, Norway) were coated with ADA2 protein, according to the manufacturer’s instructions. Specifically, the coating process involved incubating the beads overnight at 37°C with 60 µg of ADA2 protein and 80 µL (2.4 mg) of beads in 200 µL of 75 mM borate buffer (pH 9.5) containing 75 mM ammonium sulfate. The coated beads were subsequently blocked using PBS (pH 7.4) containing 0.5% (w/v) BSA for 1 h at 37°C, followed by a single wash with PBS (pH 7.4) containing 0.1% (w/v) BSA. Finally, the beads were dissolved in wash buffer to achieve a concentration of 20 mg/ml.

B. Selection of ADA2-specific scFv antibodies by phage display

Antibodies against ADA2 were enriched from a synthetic human antibody library using phage display technique. For selection, two libraries, scFvP (size 1 × 10^10^) and scFvM (size 6 × 10^9^), were mixed (19). Both libraries were in the phagemid vector pEB32x (19), which uses a truncated p3 coat protein to display scFv on the surface of the filamentous phage M13. In the first selection round, 2.4x10^12^ library phages (mixture of scFvP and scFvM libraries) in 2.5 mL of 50 mM Tris-PBS pH 7.0, 150 mM NaCl, 1% (w/v) BSA, 0.05% (v/v) Tween20 were mixed with 50 µL (1 mg) of ADA2 coated beads and incubated for 2h, at room temperature in rotation. Beads were then collected with a magnet and washed twice with 1 mL of 50 mM Tris-PBS pH 7.0, 1% (w/v) BSA, 0.1% (v/v) Tween20, followed by one wash with 1 mL of 50 mM Tris-PBS pH 7.0, 0.1% (v/v) Tween20. To elute the bound phages, the beads were resuspended in 100 µL TBS (50 mM Tris-HCl pH 7.5, 150 mM NaCl) and 100 µg/ml trypsin (Sigma, USA) and incubated for 30 min at room temperature. Next, 100 µL of 100 µg/ml soybean trypsin inhibitor (Sigma, USA) in TBS was added. E. coli XL1-Blue cells were infected with the eluted phages, and the phages were amplified using the VCS M13 helper phage (Stratagene, USA) as described previously (20). The subsequent two selection rounds were carried out in a similar way, but fewer phages (10^11^ and 10^10^ phages in 1 mL, respectively) were used, and the binding time was 1 h.

### 96-well plate phage culture for screening

E. coli XL1-Blue cells were infected with the 3^rd^ selection round phages. Gradual dilutions of the infected cells were grown at 37°C on LA plates (tryptone 10 g/L, yeast extract, 5 g/L, NaCl 5 g/L, and agar 15 g/L) with 25 µg/mL chloramphenicol, 12,5 µg/mL tetracycline, and 0.5% glucose to obtain separated colonies. Each well of a 96-well V-bottom-shaped plate containing SB medium, 10 µg/mL tetracycline, 25 µg/mL chloramphenicol, and 0.05% glucose was inoculated with an individual colony. Phage particles were produced using the VCS M13 helper phage (Stratagene, USA). Phages were screened for desired binding activity using Maxisorp plates (Nunc, Denmark) coated with ADA2. The coating was carried out overnight at room temperature with 1 µg/mL ADA2 in 50 mM Tris-HCl, 150 mM NaCl, 0.02% NaN_3_ at 100 µL/well. The coated plates were blocked with 1% bovine serum albumin (BSA) in the same buffer solution. For the detection of phage particles in the screening assay, Eu-N1-labelled anti-phage Mab was used, as previously described (20).

### scFv expression and purification

The genes encoding the most promising scFv clones were digested from the phagemid vector with SfiI and cloned into the pAK600H vector (21), followed by transformation into E. coli XL1-Blue cells. Five milliliters of SB/Amp-Tet-Glu medium (Tryptone 30 g/L, yeast extract 20 g/L, MOPS 10 g/L, 100 µg/ml Ampicillin, 12,5 µg/mL tetracycline, and 0.5% glucose) were inoculated with individual colonies and grown at 37°C. The following day, the preculture was diluted 100-200 times with fresh SB/Amp-Tet medium (without glucose) and grown until the OD600 reached 0.6. scFv production was induced using 200 µg/L IPTG. The induced cultures were grown overnight at 20°C. The next day, the bacterial cells were centrifuged, resuspended in IMAC30 (20 mM phosphate buffer, 150 mM NaCl, 30 mM imidazole, pH 7.4), and sonicated. Purification was performed using Nickel Sepharose HiTrap columns (GE Healthcare). IMAC30 was used as the binding/wash buffer, and IMAC300 (20 mM phosphate buffer, 150 mM NaCl, 300 mM imidazole, pH 7.4) was used to elute the bound proteins. The buffer was changed to TSA using GE desalting columns (Sigma Aldrich).

### ELISA with scFv-AP

Maxisorp plates (Nunc, Denmark) were coated with 5 µg/mL anti-hADA2 rabbit polyclonal antibodies (22) in PBS at +4°C overnight. The next day, the plates were washed three times with PBS and 0.05% Tween 20 and blocked with 2% BSA in PBS at room temperature for 2 h. After aspiration of the blocking buffer, the plates were stored at +4°C for three months. Next, 100 µL of the samples and hADA2 dilutions in 0.5% BSA and PBS were added to the wells and incubated for 2 h at room temperature. The plates were then washed three times with PBS and 0.05% Tween 20 and incubated for 1 h with 100 µL of 1 µg/mL H11 scFv-AP at room temperature. The plates were then washed four times with PBS containing 0.05% Tween 20. Then, 100 µL of 5 mM para-nitrophenyl phosphate (Thermo Fisher) in 50 mM Tris (pH 9), 200 mM NaCl, and 1 mM MgCl_2_ was added to each well and incubated at 37°C for 5 h. Optical density at 405 nm was determined using a Synergy H4 microplate reader (BioTek).

### Statistical analysis

Continuous and categorical variables are expressed as medians (25 and 75 percentiles) and percentages, respectively. For between-group comparisons, either the Kruskal-Wallis or Fisher’s exact test was used, as appropriate. Correlations were assessed using Spearman’s correlation coefficient. Receiver operating characteristic (ROC) curves were constructed to illustrate the diagnostic accuracy of ADA and its isoenzymes. The measures of test efficacy included sensitivity, specificity, and likelihood ratios. The level of significance was set at p<0.05 (two-tailed). Data were analyzed using a statistical software package (SPSS version 18.0; SPSS Inc., Chicago, IL, USA).

## Results

### Selection, expression, and purification of sc-anti-ADA2 alkaline phosphatase fusion protein

**Figure 1:**
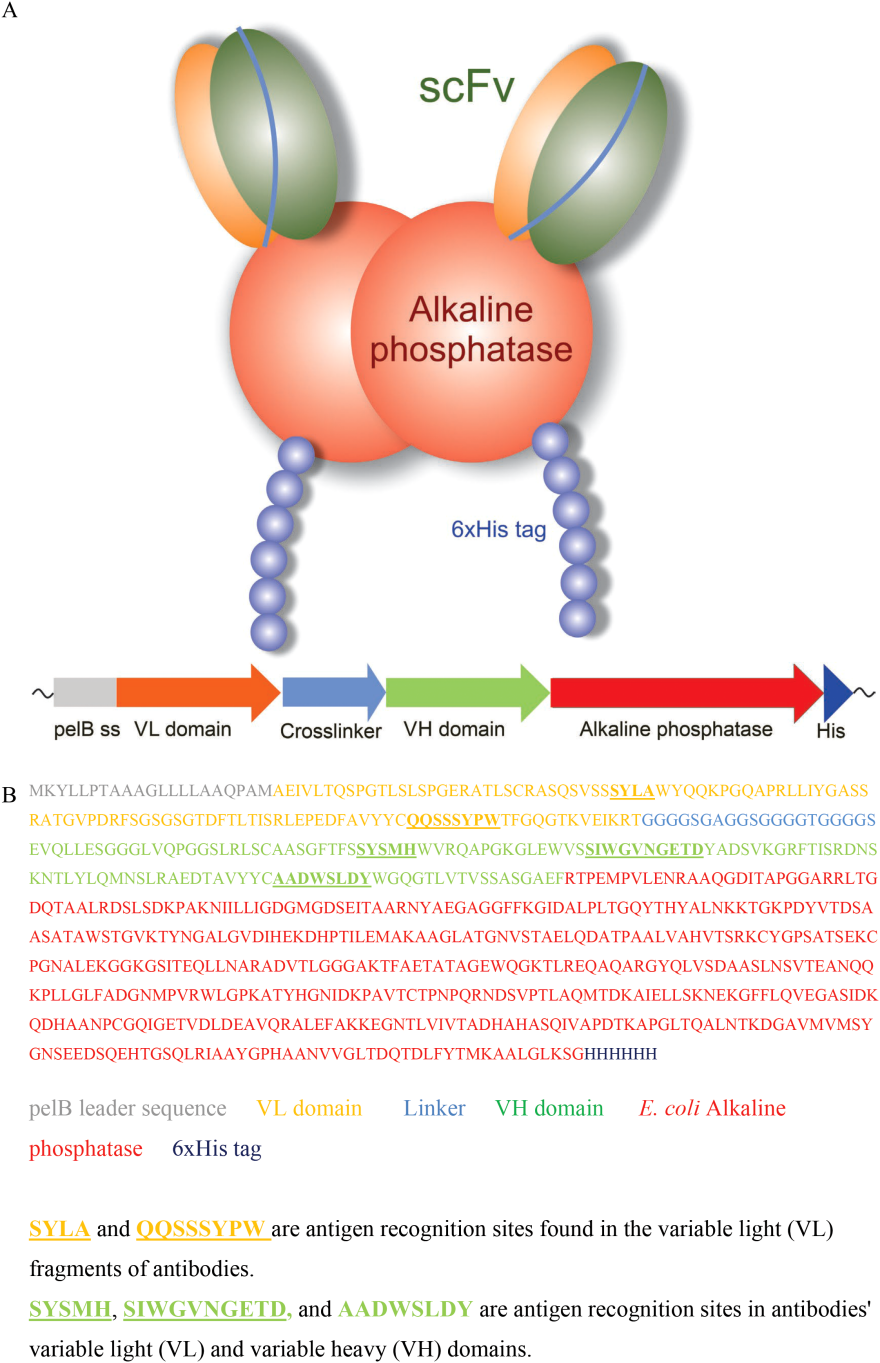
Anti-ADA2 scFv-AP structure **(A)**, gene composition, and amino acid sequence **(B)**.

ADA2 specific antibodies were enriched from synthetic single-chain antibody fragment phage libraries (19, 20) using phage display. The selections were performed against human ADA2 produced in S2 insect cells (22) covalently bound to magnetic beads. After each panning round, the phage libraries were tested for their ability to bind to ADA2 in a phage immunoassay, where phage binding to ADA2 coated 96-well plates was detected via time-resolved fluorescence spectrocopy using anti-phage Eu-labeled antibodies. After three selection rounds, when a significant increase in the immunoreactivity of the phage was observed, 95 bacterial colonies were used to inoculate cultures on a 96-well plate to produce individual scFv-phage clones. When tested for their ability to bind to ADA2 using an immunoassay, 15 phage clones were found to selectively bind to the enzyme. The anti-ADA2 single-chain antibody encoding genes were cloned from the phagemid into the pAK600H vector for the expression of scFv as a fusion with bacterial alkaline phosphatase (scFv-AP). Recombinant anti-ADA2 scFv-AP-fused proteins were overexpressed in *E. coli* and purified using Ni-NTA Chelating Sepharose. The antibodies were tested for their ability to bind to ADA2 using biolayer interferometry. The anti-ADA2 scFv-AP fusion proteins (**Figure 1**) with the highest apparent dissociation constant (Kd<10 nM) were selected for testing in the ADA2 ELISA.

### Application of sc-anti-ADA2 alkaline phosphatase fusion protein for the sandwich ELISA

**Figure 2:**
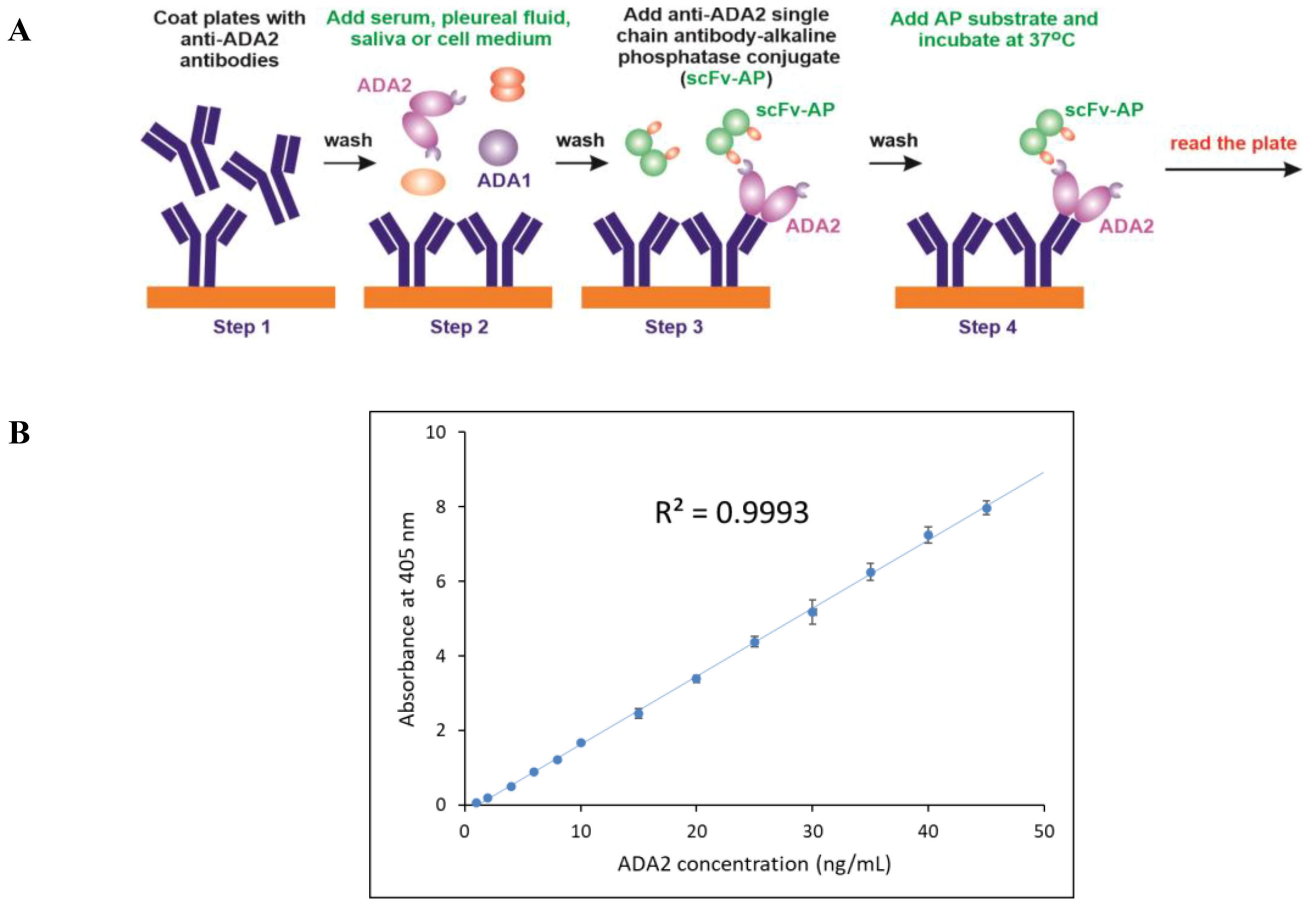
Anti-ADA2 scFv-AP ELISA **(A)** Schematic representation of the four major steps of the anti-ADA2 scFv-AP ELISA. Step 1: Coating a 96-well plate with polyclonal anti-ADA2 antibodies. Step 2: Specific binding of ADA2 to anti-ADA2 polyclonal antibodies. Step 3: Binding of anti-ADA2 scFv-AP to ADA2. Step 4: Detection of anti-ADA2 scFv-AP bound to ADA2 using an alkaline phosphatase (AP) substrate. **(B)** A typical standard curve showing a linear dependence between ADA2 concentration and absorbance at 405 nm after incubation with the alkaline phosphatase substrate p-nitrophenyl phosphate (pNPP).

For the ELISA, 96-well high-binding plates were coated with anti-ADA2 rabbit polyclonal antibodies (23) (**Figure 2A**, step 1). The second step involved standard dilutions of recombinant ADA2 and clinical samples with unknown ADA2 concentrations. After incubation and washing of the unbound proteins, anti-ADA2 scFv-AP was added to the plates (Step 3). The concentration of anti-ADA2 scFv-AP bound to ADA2 was determined using the alkaline phosphatase substrate p-nitrophenyl phosphate (pNPP). Following incubation, the increase in absorbance at 405 nm was measured using a microplate reader. A typical standard curve is presented in **Figure 2B**. The detection limit for ADA2 concentration in clinical samples was 1 ng/ml. Anti-ADA2 scFv-AP was remarkably stable, providing reliable and reproducible results after 5 years of storage (**Supplementary Figure S1**).

### Analysis of pleural effusions of the patients with TB

**Figure 3:**
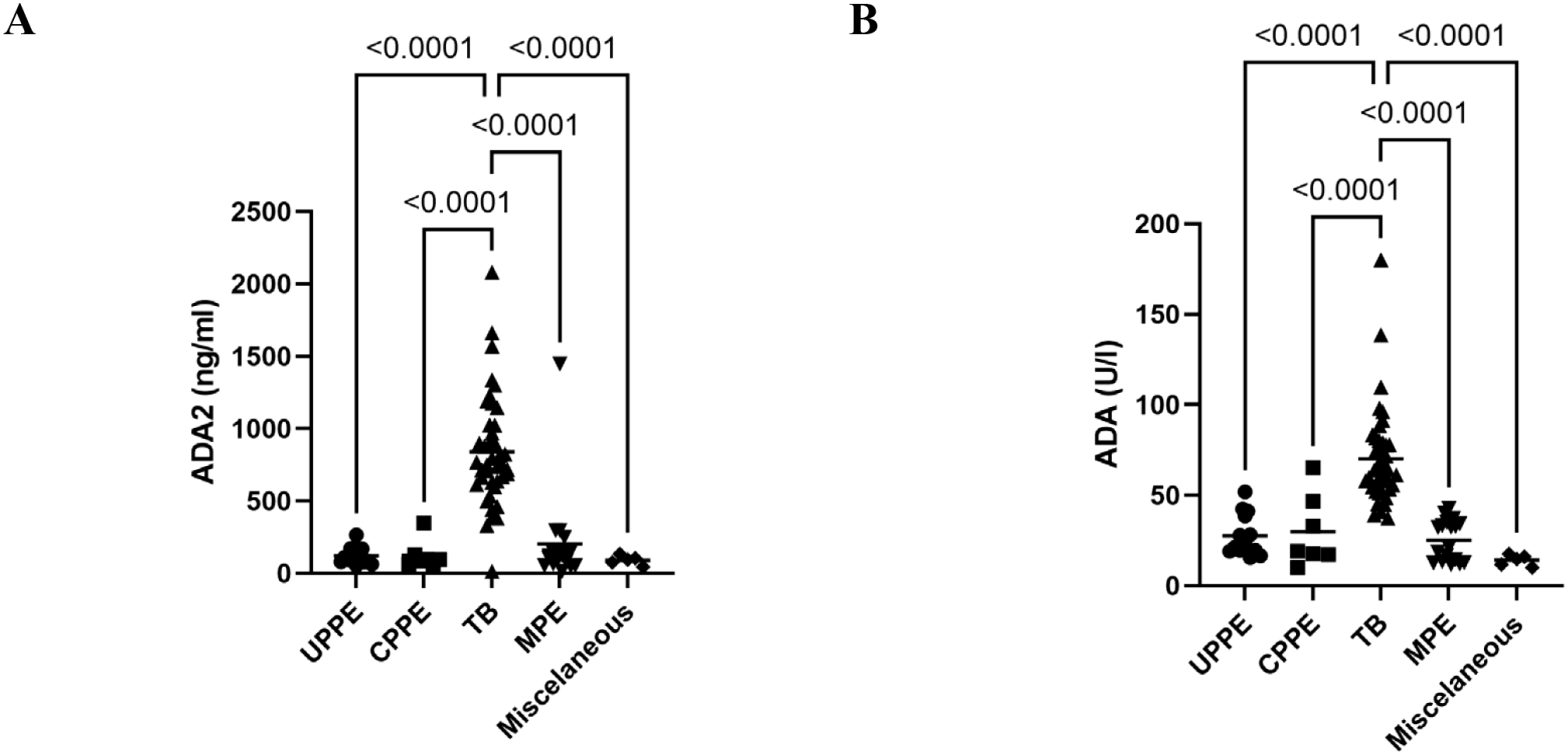
Distribution of pleural fluid ADA2 isoenzyme concentration and total ADA activity in the different diagnostic groups. **(A)** ADA2 concentrations in pleural fluid from different diagnostic groups were obtained using ADA2 ELISA with anti-ADA2 scFv-AP. **(B)** Total ADA activity in various diagnostic groups was determined using a colorimetric assay. ADA, adenosine deaminase; CPPE, complicated parapneumonic effusions; MPE, malignant pleural effusion; TB, tuberculosis; UPPE, uncomplicated parapneumonic effusions.

**Figure 4:**
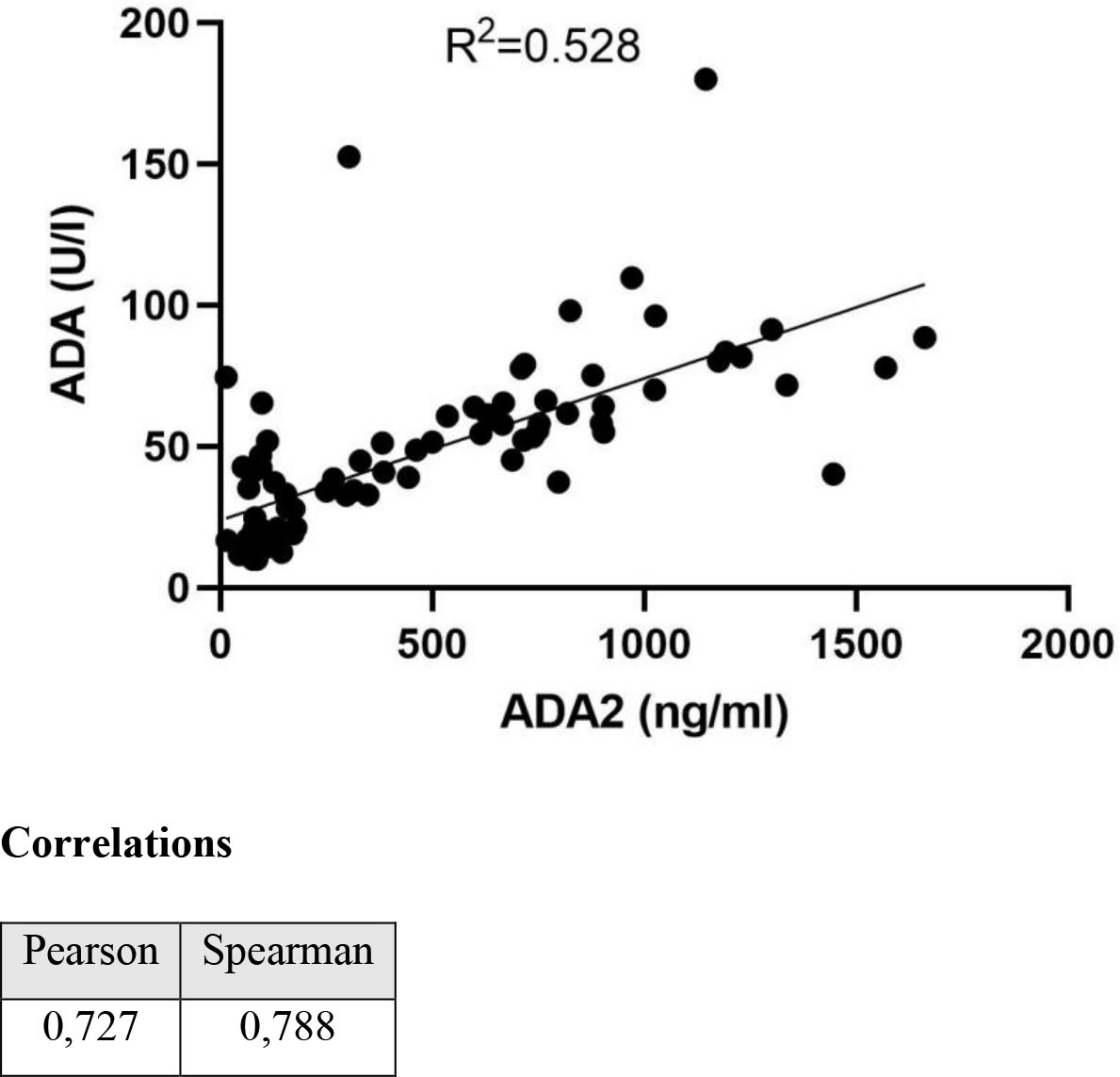
Correlation between total ADA and ADA2 isoenzyme.

**Figure 5:**
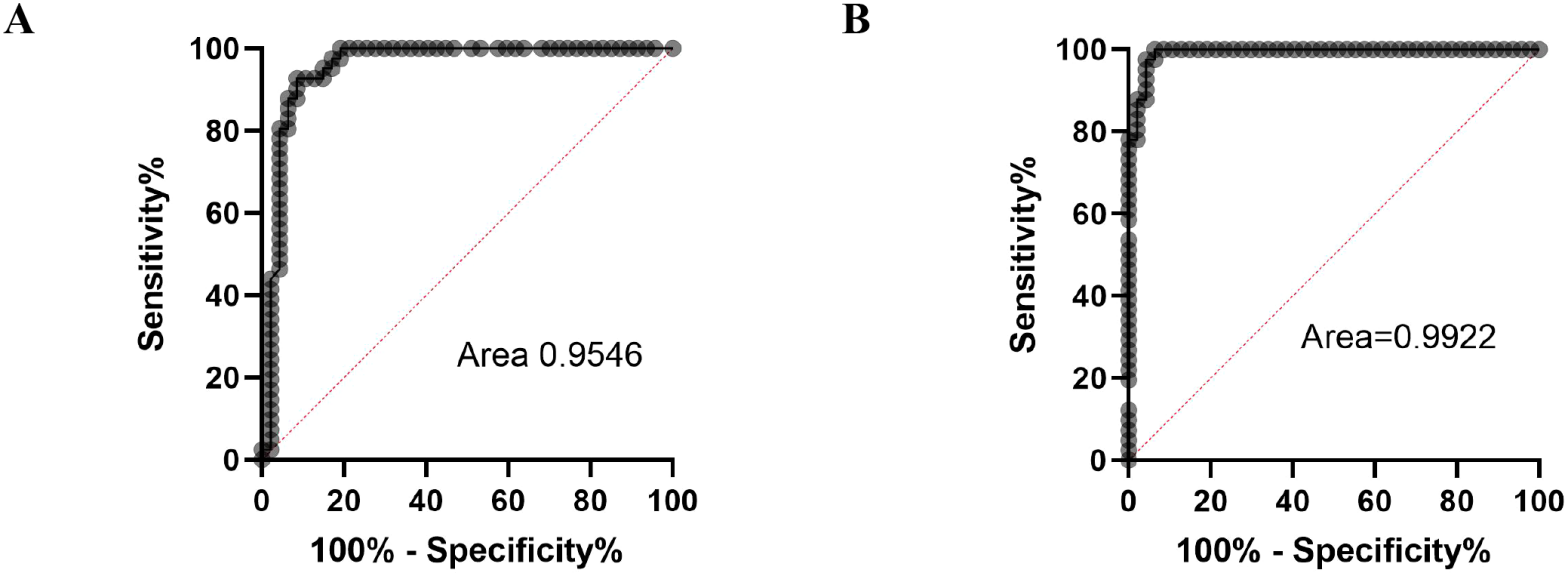
ROC curves for total ADA activity **(A)** and ADA2 concentrations **(B)**.

To validate the ADA2 ELISA with anti-ADA2 scFv-AP (**Figure 1A**), we analyzed pleural effusions from 41 patients with pleural TB and 47 with non-TB effusions. The characteristics of the study population are presented in **Table 1**. Patients with TB were considerably younger (median age 32 years) and uniformly exhibited total ADA values above the diagnostic threshold of 35 U/L, in contrast to the older age and variable total ADA levels observed in the non-TB groups. As expected, the total ADA and ADA2 concentrations were significantly higher in pleural fluid from patients with TB, both confirmed and probable cases (**Table 2**) (9). This finding suggests a robust association between TB and increased ADA2 secretion. The distribution of the ADA measurements is shown in **Figure 3**. Panel A shows that ADA2 concentrations segregate TB from non-TB effusions, whereas Panel B shows a broader overlap in the total ADA activity. This visual distinction underscores the enhanced discriminatory capability of ADA2. Additionally, **Figure 4** demonstrates a strong positive correlation between ADA2 and total ADA (Pearson’s r = 0.727; Spearman’s r = 0.788), confirming that ADA2 constitutes a significant component of total ADA but provides enhanced diagnostic specificity when measured selectively. The receiver operating characteristic (ROC) analysis in **Figure 5** further supports the utility of the ADA2 assay, as its ROC curve shows a higher area under the curve than that of the total ADA assay, indicating improved accuracy in distinguishing between tuberculous and non-tuberculous effusions. In terms of assay specificity, **Table 3** shows that several non-TB specimens (e.g., 4 of 14 UPPE and 2 of 8 CPPE cases) exhibited false-positive total ADA values, while the corresponding ADA2 levels remained low, except for one CPPE. This reduction in false positives is critical for achieving diagnostic precision. Complementing these findings, **Table 4** presents the diagnostic performance of the ADA2 assay: using a cutoff of 300 ng/mL, the assay achieved a sensitivity of 98% and specificity of 91%, with a positive likelihood ratio (LR+) of 11.5 and a negative likelihood ratio (LR–) of 0.03. These metrics indicate that a positive ADA2 result increases the pre-test probability of TB by approximately 50%, whereas a negative result virtually rules out the disease. Finally, **Table 5** focuses on malignant pleural effusions and shows that, although most malignancies exhibit low ADA2 concentrations, lymphoma cases have significantly elevated ADA2 levels (median, 300 ng/mL), a finding that warrants cautious interpretation when malignancy is considered.

**Table 1:** Study population.

	N	Age, yrs	Male^2^	Total ADA> 35 U/L
UPPE	14	77 (56-86)	7 (50)	4 (29)
CPPE	8	62 (50.74)	4 (50)	2 (25)
Confirmed TB	13	31 (26-41)	9 (69)	13 (100)
Probable TB	28	32 (25-45)	21 (75)	28 (100)
All TB	41	32 (26-43)^1^	30 (73)	41 (100)
Malignancy^3^	20	77 (66-83)	15 (75)	5 (25)
Other effusions^4^	5	77 (44-87)	4 (80)	0
Total	88	55 (32-43)	60 (68)	52 (59)

Data are expressed as medians (interquartile range) or numbers (frequency), as appropriate.

^1^Significantly lower than the respective values in other groups by the Kruskal-Wallis test (p<0,01).

^2^No significant differences between groups by Fisher’s test (p=0,34).

^3^Lung cancer 7 (3 adenocarcinomas, 2 squamous, 2 small-cell); unknown origin 5; lymphoma 4; breast 1; ovary 1; mesothelioma 1; sarcoma 1.

^4^Acute pericarditis 2, heart failure 1, Dressler syndrome 1, idiopathic 1.

ADA, adenosine deaminase; CPPE, complicated parapneumonic effusions; UPPE, uncomplicated parapneumonic effusions; TB, tuberculosis.

**Table 2:** Pleural ADA and ADA2.

	n	Total ADA, U/L	ADA2, ng/mL
UPPE	14	23 (19-39)	95 (80-171)
CPPE	8	20 (17-43)	90 (68-122)
Confirmed TB	13	61 (51-80)	714 (499-1187)
Probable TB	28	64 (55-80)	782 (639-1010)
All TB	41	64 (54-80)^1^	751 (622-1025)^1^
Malignancy	20	32 (14-35)	130 (71-285)
Other effusions	5	15 (13-17)	95 60-119)

Data are presented as median (interquartile range).

^1^Significantly higher than the respective values in other groups by Kruskal Wallis test. (p<0.01).

ADA, adenosine deaminase; CPPE, complicated parapneumonic effusions; UPPE, uncomplicated parapneumonic effusions; TB, tuberculosis.

**Table 3:** False elevated pleural fluid ADA values.

	Total ADA > 35U/L	ADA2 > 300 ng/mL
UPPE	4/14	0/14
CPPE	2/8	1/8
Confirmed TB	13/13	13/13
Probable TB	28/28	27/28
Malignancy	5/20^2^	3/20^1^
Other	0/5	0/5

^1^lymphoma 2, and small-cell lung cancer 1.

^2^lymphoma 2, unknown adenocarcinoma origin, breast carcinoma, and small-cell lung cancer (1 each).

ADA, adenosine deaminase; CPPE, complicated parapneumonic effusions; UPPE, uncomplicated parapneumonic effusions; TB, tuberculosis.

**Table 4:** Diagnostic accuracy of pleural fluid ADA and ADA2 scFv-AP in diagnosing tuberculous pleural effusion.

	Sensitivity, % (95%CI)	Specificity, % (95%CI)	LR+ (95%CI)	LR- (95%CI)
ADA2 >300 ng/mL	98 (87-100)	91 (80-97)	11.5 (4.5-29.3)	0.03 (0.004-0.19)
ADA2 >400 ng/mL	90 (77-96)	98 (89-100)	42.4 (6.1 –295.6)	0.1 (0.04-0.25)
ADA2 > 500 ng/mL	90 (77-96)	98 (89-100)	42.4 (6.1-295.6)	0.1 (0.04-0.25)
ADA2 > 600 ng/mL	78 (63-88)	98 (89-100)	36.7 (5.2-257)	0.22 (0.13-0.4)
Total ADA >35 U/L	99 (90-100)	76 (62-86)	4,1 (2.5-6.8)	0.02 (0.001-0.25)
Total ADA >40 U/L	93 (81-97)	83 (70-91)	5.4 (2.9-10.3)	0.09 (0.03-0.26)

ADA, adenosine deaminase.

**Table 5:** Pleural fluid ADA2 and total ADA levels in malignant pleural effusions.

	Total ADA, U/L	ADA2, ng/mL
Lung	32 (12-34)	83 (52-249)
Unknown origin	17 (13-35)	127 (53-220)
Lymphoma	37 (32-124)	300 (189-1160)
Miscellaneous	20 (15-32)	115 (77-131)

ADA, adenosine deaminase.

## Discussion

ADA plays a crucial role in differentiating tuberculous from non-tuberculous pleural effusions, particularly in regions with high TB prevalence. Among its isoenzymes, ADA2 is the primary contributor to increased activity in TB effusions, and is mainly secreted by stimulated macrophages. This increase is consistent with previous findings that ADA2 mRNA levels increase upon exposure to mycobacterial antigens (24).

In this study, we developed an assay that selectively measures human ADA2 levels using polyclonal capture antibodies and anti-ADA2 scFv-AP for detection. Unlike traditional activity-based assays, our method provides concentration values that are unaffected by pH or substrate levels, enabling standardized interpretation across various clinical settings. Importantly, ADA2 concentrations can be converted into activity units under specific assay conditions, and a cutoff of 300 ng/mL corresponds to 34.2 U/L—closely matching the 35 U/L threshold of total ADA assays (25).

Our results showed that ADA2 levels in pleural effusions from patients are significantly higher than those in non-TB exudates. This distinction is visually and statistically clear (**Figure 3A**), whereas the overlap in total ADA values (**Figure 3B**) highlights the increased specificity of ADA2. The strong linear correlation between total ADA and ADA2 (**Figure 4**) confirms ADA2 as a key contributor but also emphasizes that selective quantification improves the diagnostic accuracy.

Receiver operating characteristic (ROC) analysis further supports ADA2’s clinical importance. Using a cutoff of 300 ng/mL, the assay achieved 98% sensitivity and 91% specificity, with a negative likelihood ratio of 0.03 and a positive likelihood ratio of 11.5 (**Table 4**). These figures indicate a high chance of ruling out TB in negative cases and a significant increase in diagnostic confidence when the results are positive.

ADA2 measurement also lowers the number of false positives. Several non-TB cases exceeded the total ADA threshold; however, their ADA2 levels remained below the diagnostic levels (**Table 3**). This improvement is clinically significant, especially for distinguishing TB from complex parapneumonic effusions or certain cancers.

Interestingly, among malignant pleural effusions, lymphoma exhibited significantly higher ADA2 concentrations (**Table 5**). While most malignancies show low ADA2 levels, this exception emphasizes the need for careful interpretation, especially when lymphoma is suspected. More research is needed to assess the potential of the lactate dehydrogenase (LDH)/ADA2 ratio in pleural fluid for distinguishing between tuberculous and parapneumonic effusions in cases with diagnostic uncertainty. This idea is supported by studies demonstrating the usefulness of the total LDH/total ADA ratio in such cases. (26).

Despite these promising findings, there are some limitations. The single-center retrospective design and modest sample size require wider validation. In addition, assay standardization across laboratories remains a concern. Nonetheless, the ADA2 assay offers a significant advance in TB diagnostics, providing enhanced specificity, a streamlined workflow, and adaptability to other ADA2-related conditions, such as DADA2, HIV, and cancer.

## Data Availability

The raw data supporting the conclusions of this article will be made available by the authors, without undue reservation.
