# Supplementary material for: Developing and validating anti-ADA2 single-chain antibodies coupled to alkaline phosphatase for diagnosing pleural tuberculosis
Source: Front Immunol. 2025 Aug 14;16:1646134. doi: 10.3389/fimmu.2025.1646134 (PMC12390807; doi:10.3389/fimmu.2025.1646134)
Supplement: Supplementary file 1 [file SupplementaryFile1.docx]

**Supplementary material**


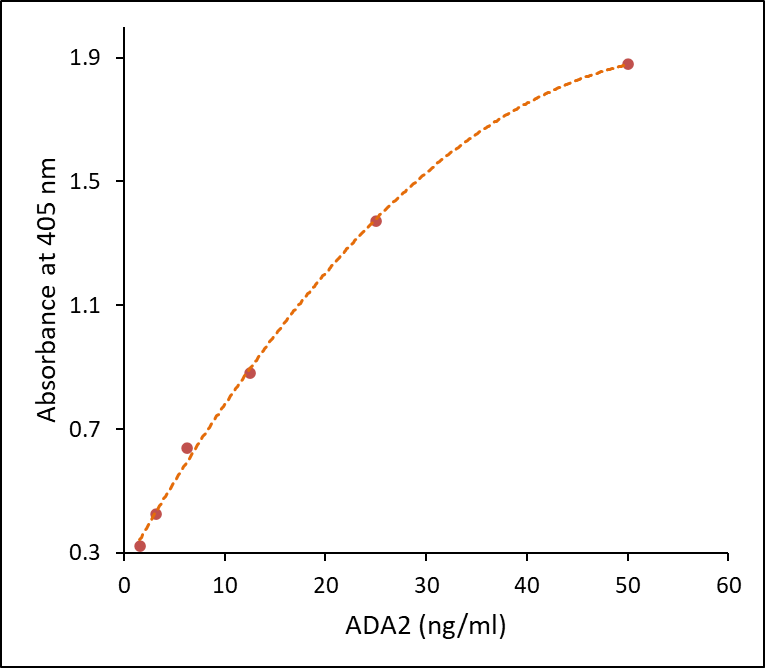


**Figure S1**. A standard curve was obtained after 5 h of incubation with the alkaline phosphatase substrate pNPP. The anti-ADA2 scFv-AP was stored at 4 °C for 5 years.
